# Supplementary material for: Potential Factors Influencing Complete Functional Recovery in Traumatized Unowned Cats with Orthopedic Lesions—A Cohort Study
Source: Vet Sci. 2024 Feb 1;11(2):59. doi: 10.3390/vetsci11020059 (PMC10892440; doi:10.3390/vetsci11020059)
Supplement: Supplementary file 1 [file vetsci-11-00059-s001.zip › vetsci-2754896-supplementary.pdf]

Supplementary Files

Supplementary Table S1. Signalment and anamnestic data for each patient subdivided in the four outcome classes (CFR vs. no-CFR vs. death from panleukemia vs. death from comorbidities).

Patient #	Age	Sex	BW	BCS	Time to referral
			**CFR**		
**CFR group**	24 (1-144)	FI 9 (26.5%)<br>FS 2 (6%)<br>MI 14 (41%)<br>MN 9 (26.5%)	1.7 (0.7-5.0)	3/9 6 (18%)<br>4/9 17 (50%)<br>5/9 5 (15%)<br>6/9 5 (15%)<br>7/9 1 (2%)	3 (0-60)
1	2	FI	1.4	4	15
2	18	MI	3.0	4	1
3	3	MI	1.0	4	10
4	24	MI	5.0	5	1
5	60	MN	4.0	4	30
6	24	MI	3.6	4	20
7	9	FI	3.0	5	8
8	96	MI	3.4	4	2
9	48	MN	4.0	6	1
10	96	MI	4.2	4	1
11	96	MN	2.8	3	60
12	24	FI	3.6	4	0
13	12	FS	3.4	6	10
14	120	MN	3.5	3	1
15	96	MN	3.3	3	20
16	48	MI	4.0	5	6
17	1	MI	0.7	4	2
18	2	MI	0.8	3	0
19	48	MN	3.3	4	8
20	24	FS	3.0	4	60
21	30	FI	2.7	3	1
22	3	MI	1.0	4	1
23	18	MN	3.0	3	43
24	24	FI	5.0	6	4
25	36	FI	3.3	4	1
26	72	FI	2.8	4	0
27	3	MI	1.1	4	1
28	144	FI	4.8	7	0
29	34	MN	3.4	5	3
30	4	FI	1.2	4	3
31	4	MI	1.8	5	7
32	60	MN	4.5	6	19
33	5	MI	1.1	4	1
34	12	FI	2.9	4	10
			**No-CFR**		
**No-CFR group**	15 (2-180)	FS 1 (17%)<br>MI 5 (83%)	2.9 (1.0-3.2)	4/9 6 (100%)	2 (0-21)
35	3	MI	1.0	4	21
36	2	MI	1.0	4	0
37	6	MI	2.7	4	3
38	24	MI	3.1	4	21

39	48	MI	3.1	4	1
40	48	MI	4.8	6	2
**No-CFR Comorbidities**					
**Comorbidities group**	24 (9-96)	FS 1 (33.3%)<br>MI 1 (33.3%)<br>MN 1 (33.3%)	3.6 (2.0-4.1)	4/9 3 (100%)	4 (0-5)
41	180	FS	3.2	4	1
42	96	MN	4.1	4	5
43	9	MI	2.0	4	4
**No-CFR Panleukopenia**					
**Panleukopenia group**	3 (2-3)	FI 2 (40%)<br>FS 1 (20%)<br>MI 2 (40%)	1.0 (0.9-1.2)	4/9 2 (40%)<br>5/9 3 (60%)	1 (0-10)
44	3	MI	1.0	5	1
45	3	MI	1.0	4	10
46	2	FI	1.0	5	0
47	2	FI	0.9	4	7
48	3	FS	1.2	5	1

Median (range) and number (%) of the variables summarized have been reported for the single groups in the first row of each group. CFR: complete functional recovery; age is expressed in months; sex: FI, intact female; FS, spayed female; MI, intact male; MN, neutered male; BW: body weight expressed in Kg; BCS: body condition score—expressed in a nine-point scale [12]; Time to referral is defined as the time elapsed from the first identification from the public veterinary service of a symptomatic unowned cat and the visit at the veterinary teaching hospital of the university of Milano expressed in days.

Supplementary Table S2. Clinical data for each patient subdivided in the four outcome classes (CFR vs. no-CFR vs. death from panleukemia vs. death from comorbidities).

Patient #	AOL	COL	Single/Poli	Location	Concomitant	Comorbidities	Time to therapy	Therapy	Complications	Hospitalization time
						**CFR**				
1	none	fracture	single	pelvis	none	megacolon	22	S -O	none	10
2	luxation	none	poli	head, thorax, abdomen	diaphragmatic hernia	pectus excavatum	1	S -I	SSI	5
3‡	fracture, luxation	none	poli	head, forelimb	none	chronic rhinitis	20	S -E	none	8
4	luxation	none	single	head	none	none	7	S -I	none	3
5	fracture, luxation	none	single	forelimb	none	none	31	S -I	none	30
6	fracture	none	single	hindlimb	none	none	21	S -I	none	20
7	fracture, luxation	none	poli	spine, pelvis	none	giardiasis	10	S -I	none	20
8	fracture	none	poli	hindlimb, abdomen	urethral rupture	none	2 – 18*	S -I	none	36
9‡	luxation	none	single	head	none	none	6	S -I	none	8
10‡	fracture, luxation	none	single	head	head trauma	none	8	S -I	none	30
11	none	fracture, luxation	single	hindlimb	cutaneous sore	none	60	S -O	none	3
12‡	fracture, luxation	none	poli	head, abdomen	head trauma, fetal death	none	0	S -I	none	15
13	fracture	none	single	hindlimb	none	none	12	S -I	none	4
14	fracture, luxation	spondylarthrosis	poli	spine, pelvis	urinary retention	none	1	C	none	11
15	none	malunion	single	forelimb	none	none	25	S -E	none	15
16	luxation	none	poli	spine, hindlimb	skin wound	none	6	S -O	none	8
17	fracture	none	single	hindlimb	none	none	2	C	none	1

18	fracture, luxation	none	poli	head, hindlimb	head trauma	none	0	C	none	12
19‡	fracture, luxation	malunion	poli	head, forelimb, hindlimb	none	none	8	S -I	implant failure	19
20	none	fracture	single	pelvis	none	none	60	C	none	1
21	fracture	none	poli	pelvis, hindlimb	none	pyometra	24	S -I	none	30
22	fracture	none	single	hindlimb	none	none	9	S -O	none	10
23	none	fracture, malunion	poli	pelvis, hindlimb	skin wound	none	60	S -I	none	20
24	fracture	none	poli	pelvis, hindlimb	none	none	5	S -E	none	3
25	luxation	none	single	forelimb	none	none	2	S -I	implant failure	27
26	fracture	none	single	pelvis		none	10	S -I	none	30
27	fracture	none	poli	spine, pelvis, thorax, abdomen	diaphragmatic hernia, body wall hernia	none	1	S -O	none	10
28‡	fracture	none	single	head	head trauma	none	7	S -I	none	14
29	fracture	none	single	head	head trauma	none	5	S -I	none	7
30	fracture	none	poli	head, hindlimb	head trauma	none	5	S -I	none	10
31	fracture	none	single	forelimb	skin wound	none	8	S -O	none	8
32	luxation	none	poli	pelvis, hindlimb	skin wound	none	19	S -O	SSI	20
33	fracture	none	single	pelvis	none	flea infestation	1	C	none	3
34	fracture	none	single	hindlimb	none	hyperthermia	18	S -E	none	40
					**No-CFR**					
35	fracture, luxation	none	poli	spine, pelvis	none	megacolon, urinary retention	21	S -I	implant failure	20
36	fracture	none	single	spine	none	none	0	C	none	45
37	fracture	none	single	hindlimb	none	none	6	S -E	implant failure	90

38	fracture	none	poli	hindlimb, abdomen	body wall hernia	none	31	S -O	none	18
39‡	fracture, luxation	none	poli	spine, pelvis, head	head trauma	none	3	S -I	none	30
40	fracture, luxation	none	poli	hindlimb, pelvis	none	none	7	S -I	implant failure	25
**No-CFR Comorbidities**										
41	fracture, luxation	elbows, stifle, hips osteoarthritis	single	hindlimb	none	renal failure	1	C	death	1
42	fracture	none	single	hindlimb	none	feline infectious peritonitis	5	C	death	2
43	fracture, luxation	none	poli	pelvis, hindlimb, spine	none	hypertrophic cardiomyopathy	4	C	death	12
**No-CFR Panleukopenia**										
44	fracture	none	poli	hindlimb, thorax	diaphragmatic hernia	none	2	S -I	death	10
45	fracture	none	single	forelimb	none	none	10	S -I	death	30
46	fracture	none	poli	forelimb, head	head trauma	none	3	S -I	death	14
47	fracture	none	single	spine	spinal trauma	none	7	C	death	1
48	fracture	none	poli	pelvis, thorax, abdomen	diaphragmatic hernia, body wall hernia, ureteral rupture	none	3	S -O	death	14

CFR: complete functional recovery; AOL: acute orthopedic lesion; COL: chronic orthopedic lesions; ‡: patient with esophagostomy tube placement; time to therapy is the estimated time between the first identification from the public veterinary service of a symptomatic unowned cat, and the start of the therapy at the veterinary teaching hospital of the university of Milano expressed in days; *laparotomy was performed at the time of the arrival (2 days after the trauma) at the veterinary teaching hospital after the diagnosis of uroperitoneum. Femoral osteosynthesis was performed 18 days after the trauma; therapy—C: conservative, S: surgical, -E: external fixation, -I: internal fixation, -O: other surgical procedures. SSI: surgical site infection—inflammation. Hospitalization time was defined as the time elapsed from the first visit at the veterinary teaching hospital of the university of Milano expressed in days to the hospital discharge or death.

Supplementary Table S3. Radiographic scores for each patient subdivided in the four outcome classes (CFR vs. no-CFR vs. death from panleukemia vs. death from comorbidities).

Patient n.	Skeletal s.	Thoracic s.	Abdominal s.	TTRS
**CFR**				
1	2	-	-	-
2	0	2	0	2
3	1	0	0	1
4	1	0	0	1
5	2	-	-	-
6	1	-	-	-
7	2	0	0	2
8	1	-	1	-
9	1	0	-	-
10	1	0	0	1
11	1	-	-	-
12	1	0	0	1
13	1	0	0	1
14	1	0	0	1
15	1	-	-	-
16	2	-	-	-
17	1	0	0	1
18	2	0	0	2
19	3	-	-	-
20	2	-	0	-
21	1	-	-	-
22	-	-	-	-
23	2	0	0	2
24	1	0	0	1
25	2	0	-	-
26	1	0	0	1
27	2	2	1	5
28	2	0	0	2
29	1	0	0	1
30	1	0	0	1

31	1	0	0	1
32	2	0	0	2
33	1	0	0	1
34	1	0	0	1
**No-CRF**				
35	2	0	0	2
36	2	0	0	2
37	1	0	0	1
38	1	-	1	-
39	3	0	0	3
40	2	0	0	2
**No-CFR Comorbidietes**				
41	-	-	-	-
42	1	-	0	-
43	3	0	0	3
**No-CFR Panleukemia**				
44	3	1	0	4
45	1	-	-	-
46	1	-	-	-
47	2	0	0	2
48	3	1	1	5

CFR: complete functional recovery; s.: score; TTRS: total trauma radiographic score; -: radiographs not performed.

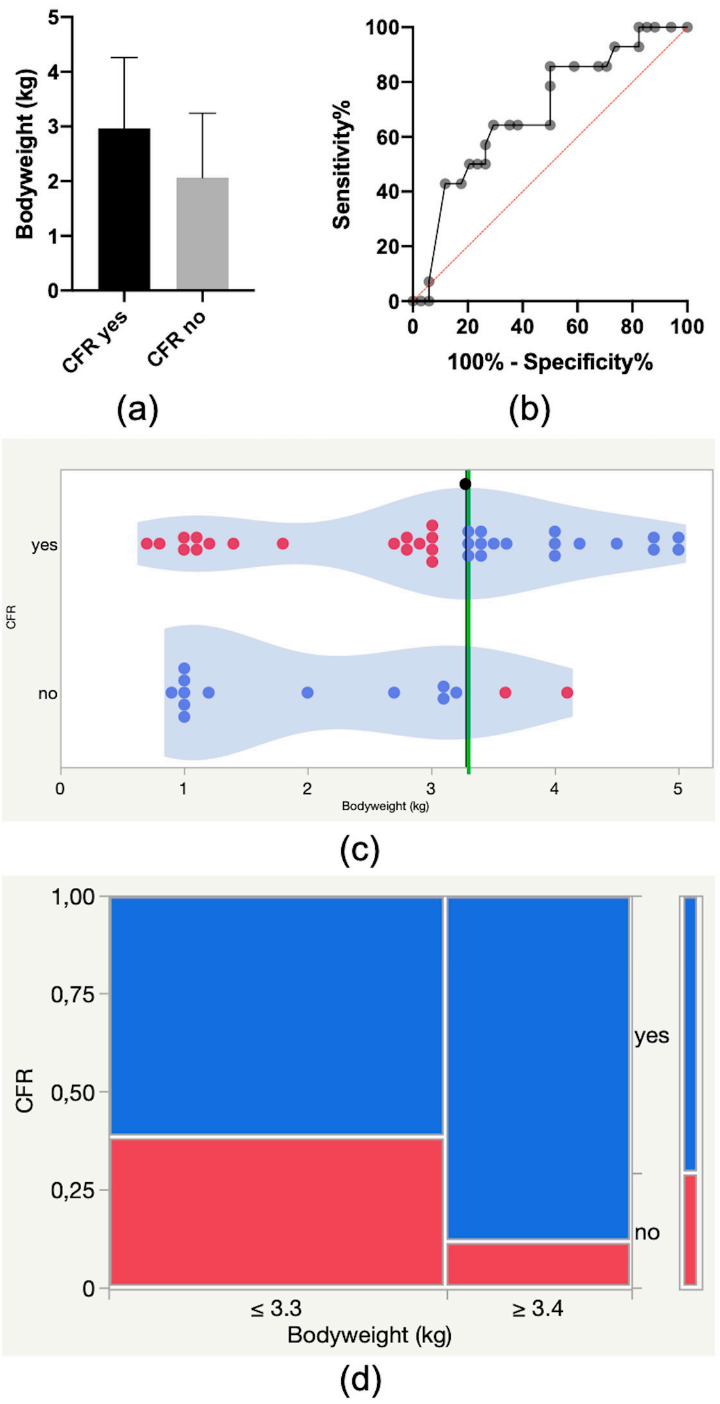

Supplementary Figure S1. Graphic representation of the two-level statistic to explore the association between body weight and CFR. In (a), body weight, represented as mean ± SD, was significantly higher in cats experiencing CFR (CFR yes) than those with no-CFR (CFR no). In (b), the ROC curve of the body weight to obtain a cut-off value. In (c), graphic representation of the cut-off value for the body weight over the plotted values in the two classes (CFR *vs*. no-CFR). In (d), graphic representation of the number of patients experiencing CFR (in blue) or not-experiencing CFR (in red) in the two body weight classes (≤ 3.3 kg on the left, ≥ 3.4 kg on the right).

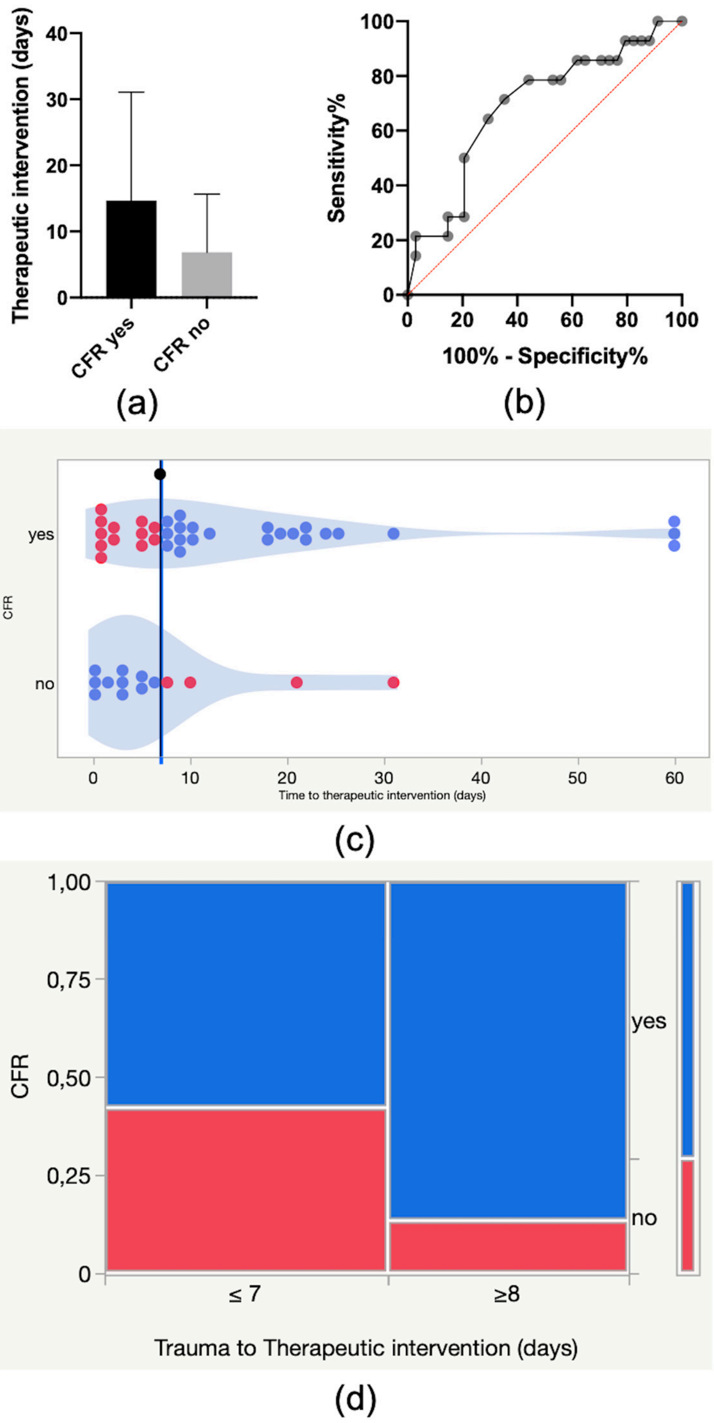

Supplementary Figure S2. Graphic representation of the two-level statistic to explore the association between the estimated time from trauma to therapeutic intervention and CFR. In (a), time to therapeutic intervention represented as mean ± SD was significantly longer in cats experiencing CFR (CFR yes) than those with no-CFR (CFR no). In (b), the ROC curve of the time to therapeutic intervention to obtain a cut-off value. In (c), graphic representation of the cut-off value for the time to therapeutic intervention over the plotted values in the two classes (CFR *vs*. no-CFR). In (d), graphic representation of the number of patients experiencing CFR (in blue) or not-experiencing CFR (in red) in the two time-to-therapeutic intervention classes (≤ 7 days on the left, ≥ 8 days on the right).
